# Correction to “In Vitro and In Planta Botanical Control of Banana Postharvest Disease Causing Fungi”

**DOI:** 10.1002/fsn3.71876

**Published:** 2026-05-18

Hossain, A., F. Akter, P. Ghosh, S. M. Naimul Islam, M. A. B. Bhuiyan, and S. S. Siddique. 2026. “In Vitro and In Planta Botanical Control of Banana Postharvest Disease Causing Fungi.” *Food Science & Nutrition* 14, no. 3: e71557. https://doi.org/10.1002/fsn3.71557.

All authors' contribution are added to the article. The author contribution section should read as below:

**Afsana Hossain:** analysis, visualization, review. **Farjana Akter:** data curation, investigation. **Pallab Ghosh:** data curation. **Shah Mohammad Naimul Islam:** formal analysis, software. **Md. Abdullahil Baki Bhuiyan:** fund acquisition, validation, review. **Shaikh Sharmin Siddique:** conceptualization, methodology, formal analysis, validation, writing – original draft.

We apologize for this error.

